# Comparison of targeted next-generation sequencing and the Xpert MTB/RIF assay for detection of *Mycobacterium tuberculosis* in clinical isolates and sputum specimens

**DOI:** 10.1128/spectrum.04098-23

**Published:** 2024-04-11

**Authors:** Hongtai Zhang, Xiaowei Dai, Peilei Hu, Lili Tian, Chuanyou Li, Beichuan Ding, Xinyu Yang, Xiaoxin He

**Affiliations:** 1 Beijing Center for Disease Prevention and Control, Beijing, China; 2 Hunan Chest Hospital, Changsha, Hunan Province, China; Université Paris-Saclay, Clamart, France

**Keywords:** tuberculosis, *Mycobacterium tuberculosis*, targeted next-generation sequencing, Xpert MTB/RIF

## Abstract

Targeted next-generation sequencing (tNGS) can be used to perform *Mycobacterium tuberculosis* (MTB) complex-specific amplification or target capture directly from sputum samples, yielding simultaneous coverage of many genes and DNA regions associated with antimicrobial resistance (AMR). Performance comparisons of tNGS and another molecular testing tool, Xpert MTB/rifampicin (RIF), have been empirical. Here, using a dilution series of a RIF-resistant clinical isolate of MTB, we found that tNGS had a slightly lower limit of bacterial detection (10^2^ CFU/mL) compared with Xpert MTB/RIF (10^3^ CFU/mL) in culture medium. However, the minimum detection limit of the *rpoB* S450L mutation in this isolate was significantly lower with tNGS (10^2^ CFU/mL) than with Xpert MTB/RIF (10^6^ CFU/mL). Sputum samples collected from 129 suspected pulmonary tuberculosis patients were also prospectively studied with the clinical diagnosis as a reference, revealing that the sensitivity of tNGS (48.6%) was higher than those of culture (46.8%), Xpert MTB/RIF (39.4%), and smear microscopy (34.9%) testing. Notably, AMR analysis of 56 MTB-positive samples as determined by tNGS revealed high mutation frequencies of 96.4%, 35.7%, 26.8%, and 19.6% in the following AMR-associated genes: *rrs*, *rpoB*, *katG*, and *pncA*, respectively. The findings of this study provide theoretical support for the differential clinical application of tNGS and Xpert MTB/RIF and suggest that tNGS has greater application value in tuberculosis drug resistance monitoring and prevention.

**IMPORTANCE:**

Targeted next-generation sequencing (tNGS) can be used to perform *Mycobacterium tuberculosis* (MTB) complex-specific amplification or target capture directly from sputum samples, yielding simultaneous coverage of genes and DNA regions associated with antimicrobial resistance (AMR). Performance comparisons of tNGS and Xpert MTB/rifampicin (RIF) have been empirical. The Xpert MTB/RIF assay is a commercial system that uses the nucleic acid amplification detection method for rapid (2 hours) diagnosis of tuberculosis (TB). The cost of the tNGS and Xpert MTB/RIF assays in this study was similar, at USD 98 and USD 70–104 per sample, respectively, but the time required for tNGS (3 days) was much longer than that required for the Xpert MTB/RIF assay. However, tNGS yielded more accurate results and a larger number of AMR-associated gene mutations, which compensated for the extra time and highlighted the greater application value of tNGS in TB drug resistance monitoring and prevention.

## INTRODUCTION

Tuberculosis (TB) is a preventable and usually curable disease. Nevertheless, in 2022, TB was the world’s second leading cause of death from a single infectious agent (after COVID-19) and caused almost twice as many deaths as HIV/AIDS. More than 10 million people continue to fall ill with TB every year (1). TB is caused primarily by the bacillus *Mycobacterium tuberculosis* (MTB), which is spread when infected people who are sick expel MTB into the air (e.g., by coughing). About a quarter of the global population is estimated to have been infected with MTB (2).

In contrast to conventional culture-based diagnosis, which takes many weeks, the advent of molecular approaches has reduced MTB diagnosis times to a few days. The use of rapid molecular diagnostic methods, such as the Xpert MTB/rifampicin (RIF) assay, can lower the likelihood of person-to-person transmission, as well as TB-related morbidity and mortality rates (3).

The Xpert MTB/RIF assay is a commercial system that uses the nucleic acid amplification detection method. It has been endorsed by the World Health Organization (WHO) for rapid (2 hours) diagnosis of TB and features a single-use disposable cartridge for fully automated sample preparation, amplification, and simultaneous detection of MTB and RIF resistance via real-time PCR (4).

Targeted next-generation sequencing (tNGS) has recently become available in China as a non-approved molecular testing tool for the detection of MTB and antimicrobial resistance (AMR) (5–7). This method offers fast, precise genetic analysis, including the ability to identify genetic mutations indicative of drug resistance in a fraction of the time required for culture-based methods. However, a comparison between tNGS and Xpert MTB/RIF for the detection of MTB and AMR in laboratory and respiratory specimens has not been reported.

The aim of this study was to comparatively evaluate the performance of tNGS and Xpert MTB/RIF for the detection of MTB and AMR in established strains and sputum specimens. Additionally, tNGS was compared with conventional testing methods for the ability to detect MTB. We anticipate that the findings of this study will be valuable for the commercial application of tNGS in the detection of MTB and AMR in the clinical setting, ultimately guiding patient treatment, and the prevention and control of MTB associated with AMR.

## MATERIALS AND METHODS

### Study design

This was a prospective, multicenter, diagnostic accuracy study. The primary objectives were to: (i) compare tNGS and the Xpert MTB/RIF assay (Cepheid, Sunnyvale, CA, USA) for the minimum bacterial detection of MTB using dilution series of clinical isolates in culture medium; (ii) compare tNGS, Xpert MTB/RIF, culture, and smear microscopy for sensitivity and specificity of MTB detection in sputum specimens; (iii) compare tNGS and Xpert MTB/RIF for sensitivity in the detection of MTB AMR mutations in sputum specimens; and (iv) analyze the gene mutations associated with AMR in samples with MTB-positivity determined by tNGS.

### Experimental design

Sputum samples were collected from 129 suspected TB patients from Beijing and Changsha, China who were treated at the Beijing Center for Disease Control and Prevention and Hunan Provincial Chest Hospital, of whom 38.8% (50/129) were from Beijing and 61.2% (79/129) were from Changsha. Clinical data collected from each patient included medical history, clinical examination findings, and available radiological and pathological results. All participants provided written informed consent. All clinical specimens were examined by acid-fast bacillus (AFB) staining, liquid medium culture, Xpert MTB/RIF assay, and tNGS.

### Specimen processing

For MTB isolates, sterile inoculation loops were used to scrape the bacteria on the inclined surface of the medium, and as many colonies as possible were transferred to a grinding bottle containing glass beads. The bottle cap was tightened and the contents were mixed on a vortex vibrator for 30–60 seconds, followed by the addition of sterile phosphate-buffered saline to adjust the turbidity to that of a 1 mg/mL (~1 × 10^−7^ CFU/mL) bacterial solution (Machellogh turbidity standard). We then prepared a 10× dilution series of the bacterial solution (10^−1^, 10^−2^, 10^−3^, 10^−4^, 10^−5^, and 10^−6^) for the following experiments, with the testing at each dilution repeated three times. The same dilution series of a culture of the isolate was tested in triplicate in parallel to evaluate the sensitivity of tNGS and Xpert MTB/RIF for the detection of MTB genomic DNA and the RIF-associated gene mutation S450L.

For sputum, we used sputum boxes to collect morning sputum of suspected TB patients, from which unpurified sputum specimens were collected for the following: clinical diagnosis using the Xpert MTB/RIF assay, smears for Ziehl-Neelsen staining, and MTB culture. The remaining sputum (~1 mL) was used for DNA extraction and stored at −80°C for tNGS testing performed within 3 days.

### AFB staining and mycobacterial culture

The NaLC-NaOH-processed sputum samples were examined for Ziehl-Neelsen staining, and the results were graded in accordance with recommendations from the Beijing Centers for Disease Control and Prevention. Specimens with an AFB grade of 1–4 were defined as smear-positive (8).

Mycobacterial cultures were prepared by inoculating 500 µL of purified sample into mycobacterial growth indicator tubes (MGIT 960 system; Becton Dickinson, Sparks, MD, USA), then were cultured for 6 weeks. Positive cultures were subjected to AFB Ziehl-Neelsen staining to confirm the presence of AFB and to rule out contamination. Positive liquid cultures were also confirmed by a TB MPB64 antigen test kit (Hangzhou Genesis Biodetection & Biocontrol Ltd., Hangzhou, China).

### Xpert MTB/RIF assays

The Xpert MTB/RIF assays were conducted in accordance with the manufacturer’s instructions, as described previously (4).

#### tNGS analysis

DNA was extracted from sputum samples (~1 mL) using the Sputum Bacterial DNA Kit [XP3170-02; Shengshizhongfang (Beijing) Bio Sci & Tech. Co., Ltd. Beijing, China], in accordance with the manufacturer’s instructions. Next, a library was constructed using the TB DNA Targeted Library Prep Kit [XP3160-03; Shengshizhongfang (Beijing) Bio Sci & Tech. Co., Ltd.], which generates a library fragment size of about 280–400 base pairs (bp). Quality control assessment of the DNA concentration and fragment size was performed using an Agilent 2100 Bioanalyzer (Agilent Technologies, Santa Clara, CA, USA). The qualified libraries were pooled, diluted to 2 nM, and then loaded onto a sequencing chip for 150 bp double-ended sequencing on the Illumina NovaSeq 6000 platform (Illumina, San Diego, CA, USA). Sequencing reads were checked using FASTQC as a primary assessment of data quality. Alignment to the laboratory reference strain H37Rv was performed using BWA and Sambamb, and mutations identified in the loci of interest were compared with a compiled library of known resistance- and sublineage-associated mutations. Low-frequency variants were identified using the GATK software, with thresholds of ≥3%, five reads, and ≥1 read in both the forward and reverse directions.

A positive result for the detection of MTB by tNGS required a minimum of 100 effective specific sequences targeting each of the 158 MTB genes screened by the assay.

### Clinical TB diagnosis

Study participants were classified as having TB (“TB”) if they met either of the following criteria: (i) positive for MTB by Xpert MTB/RIF assay, sputum culture, or smear microscopy; or (ii) clinically diagnosed pathology, i.e., no molecular or microbiological evidence, but meeting the comprehensive diagnostic criteria for TB. These criteria are as follows: (i) epidemiological risk, i.e., history of contact with TB patients; (ii) clinical manifestations with suspicious symptoms of TB, i.e., cough and sputum for 2 weeks, or sputum with blood, or hemoptysis, including systemic symptoms, such as night sweats, fatigue, intermittent or continuous afternoon low fever, loss of appetite, weight loss, and other symptoms; (iii) symptoms on chest imaging examination; (iv) pathological changes on TB pathology examination, presenting as epithelial-like granulomatous inflammation, and necrotic and non-necrotic granulomas of different sizes and numbers under light microscopy; and (v) on immunological examination, a moderately positive or strongly positive tuberculin skin test, or a positive γ-interferon release test, or an antibody-positive test for MTB.

Participants were classified as not having TB (“no-TB”) if they were negative for TB under Xpert MTB/RIF assay, sputum culture, and smear microscopy, with symptom resolution without TB treatment, or if the sputum culture grew nontuberculous mycobacteria (NTM).

### Statistical analysis

SPSS 22.0 software was used for statistical analysis. The count data are expressed as number of cases and rate. The sensitivity, specificity, and consistency rates of MTB diagnosis were calculated as follows: sensitivity = number of positive cases detected by the method/number of clinically diagnosed MTB cases × 100%; specificity = number of negative cases detected by the method/number of excluded TB cases × 100%; consistency rate = (number of true positive cases + number of true negative cases)/total number of cases × 100%. Kappa tests were performed to evaluate the consistency of the final diagnosis, with kappa values indicative of the following: 0.00–0.20, extremely low consistency; 0.21–0.40, general consistency; 0.41–0.60, moderate consistency; 0.61–0.80, high consistency; and 0.81–1.00, almost complete consistency. McNemar’s test was used to compare the paired samples with other diagnostic methods. A *P* value < 0.05 was considered to indicate a statistically significant difference.

## RESULTS

The experimental design of this study, which mainly aimed to compare two molecular testing methods, tNGS and Xpert MTB/RIF, for the detection of MTB and RIF-AMR, is shown in Table S1.

In this study, a clinical isolate (no. 013) from a patient treated in the Beijing Center for Disease Control and Prevention was cultured. RIF resistance-associated mutations were determined by Sanger sequencing of the RIF resistance-determining region (RRDR) of *rpoB* (codons 426–452) to identify the S450L mutation and by phenotypic drug sensitivity assays. We also used whole-genome sequencing (WGS) of this isolate to acquire information about all of the AMR-associated mutation sites listed in the WHO catalog (9).

The minimum detection limit of tNGS (10^2^ CFU/mL) indicated that it was slightly more sensitive than Xpert MIB/RIF (10^3^ CFU/mL) for the detection of MTB (Table 1).

**TABLE 1:** Comparison of the MTB detection limits of the Xpert MTB/RIF and tNGS methods using clinical isolate no. 13^*a*^

Concentration (CFU/mL)	Xpert MTB/RIF	tNGS
10^6^	Median/Y	Y
10^5^	Median/Y	Y
10^4^	Low/Y	Y
10^3^	Very low/Y	Y
10^2^	–^*b*^	Y
10^1^	–	–

^*a*^ Y indicates positive identification of MTB.

^*b*^ – indicates no results.

Although the Xpert MIB/RIF assay detected MTB at 10^3^–10^6^ CFU/mL, it only detected *rpoB* S450L at 10^6^ CFU/mL in the culture medium (Table 2). By contrast, tNGS identified MTB as well as S450L at 10^2^–10^6^ CFU/mL of the clinical isolate (Table 2).

**TABLE 2:** Comparison of the Xpert MTB/RIF assay and tNGS for rifampicin resistance (RIF-R) testing of clinical isolate no. 13^*a*^

Concentration (CFU/mL)	Sanger (reference)	Xpert MTB/RIF	tNGS
10^6^	Y/rpoB_p.Ser450Leu	RIF-R/Y	Y/rpoB_p.Ser450Leu
10^5^		RIF-R/not detected	Y/rpoB_p.Ser450Leu
10^4^		RIF-R/not detected	Y/rpoB_p.Ser450Leu
10^3^		RIF-R/not detected	Y/rpoB_p.Ser450Leu
10^2^		–^*b*^	Y/rpoB_p.Ser450Leu

^*a*^ Y indicates that RIF-R was detected. Sanger, Sanger sequencing.

^*b*^ – indicates no results.

The mutation information was not limited to the RRDR. Using the clinical isolate data as a reference, we determined the potential of tNGS and WGS for the identification of all 17,396 AMR-associated mutation sites in the WHO catalog. We found that, at 10^2^–10^6^ CFU/mL, tNGS identified the fabG1_c.-15C > T and fabG1_c.15C > T mutations associated with isoniazid and ethionamide, and the rpsL_p.Lys88Arg mutation was associated with streptomycin, which was consistent with the results of WGS.

We also compared tNGS and the Xpert MIB/RIF assay in a prospective study of 129 patients with suspected TB (Table S2). Basic and statistical information about the cohort is provided in Table 3. Based on clinical diagnostic judgment, 84.5% (109/129) of the patients were deemed to have TB, and 15.5% (20/129) were interpreted as not having TB. Of the 20 patients without TB, four were diagnosed with NTM disease on the basis of NTM culture positivity (Table 3).

**TABLE 3:** Background and statistical information on the cohort of patients with suspected TB

Characteristic	Number	Proportion (%)
Total	129	100.0
Age		
≤50	53	41.1
>50	76	58.9
Sex		
Male	81	62.8
Female	48	37.2
Clinical diagnosis		
Positive	109	84.5
Negative	16	12.4
NTM	4	3.1
History of TB		
Initial treatment	75	58.1
Retreatment	17	13.2
Unknown	37	28.7
Region		
Beijing	50	38.8
Changsha	79	61.2
HIV test		
Negative	129	100.0

Sputum collected from the 129 patients was used to perform tNGS, Xpert MIB/RIF assay, culture, and smear microscopy assessments (Table 4). Using the clinical diagnosis as a reference, tNGS showed the highest sensitivity (48.6%; 53/109), followed by culture (46.8%; 51/109), Xpert MTB/RIF assay (39.4%; 43/109), and smear microscopy (34.9%; 38/109). The newest diagnostic method, tNGS, had a specificity of 85% (17/20). In terms of concordance of assays, culture (55.0%; 71/129) was the highest, followed by tNGS (54.3%; 70/129), Xpert MTB/RIF assay (48.8%; 63/129), and smear microscopy (45.0%; 58/129). Of 16 sputum samples that were MTB-positive under tNGS alone, 13 patients had also been clinically diagnosed with TB (Table S2). Furthermore, there were statistical differences between each of the four methods and the clinical diagnosis (Table 4).

**TABLE 4:** Comparison of tNGS, Xpert MTB/RIF, culture, and smear microscopy assessments using the clinical diagnosis of suspected TB patients as the reference^*a*^

Method	Sensitivity (%) (95% CI)	Specificity (%) (95% CI)	Consistency rate	χ^2^	*P* value	Kappa
tNGS	48.6 (53/109) (39.0–58.3)	85.0 (17/20) (61.1–96.0)	54.3 (70/129)	7.777	*P* < 0.01	0.162
Xpert MTB/RIF	39.4 (43/109) (30.4–49.3)	100.0 (20/20) (80.0–100)	48.8 (63/129)	11.835	*P* < 0.01	0.168
Culture	46.8 (51/109) (37.3–56.6)	100.0 (20/20) (80.0–100)	55.0 (71/129)	15.476	*P* < 0.01	0.214
Smear	34.9 (38/109) (26.2–44.7)	100.0 (20/20) (80.0–100)	45.0 (58/129)	9.884	*P* < 0.01	0.142

^*a*^ Sensitivity and specificity were calculated relative to the number of cases with a clinical diagnosis of TB. McNemar’s test was used to calculate χ^2^ and *P* values; *P* < 0.01 was considered statistically significant.

Among the 129 sputum samples from patients with suspected TB, using culture positivity as a reference, the sensitivities of tNGS (76.5%; 39/51), Xpert MIB/RIF (78.4%; 40/51), and smear microscopy (72.5%; 37/51) were found to be similar (*P* = 0.458, *P* = 0.057, *P* = 0.001, respectively; Table S3). Three samples were MTB-positive under culture and tNGS assay alone, while four samples were positive under culture and Xpert MIB/RIF assay alone (Table S2). Because tNGS was not used as a clinical diagnostic method to confirm TB, our reports were provided to the three patients with culture and tNGS positivity and their doctors for subsequent observation (Table S2).

Of the 129 sputum samples, 67 samples tested positive under one or both of the molecular testing methods: 56 under tNGS, 43 under Xpert MIB/RIF, and 32 under both (Table 5). Twenty-four samples were only detected by tNGS, and 11 were only detected by Xpert MIB/RIF. The statistical difference between tNGS and Xpert MIB/RIF (McNemar’s test: *P* = 0.041, χ^2^ = 25.245) indicated that these two molecular tests cannot be used interchangeably.

**TABLE 5:** Comparison of sputum samples that tested positive for TB under tNGS and/or the Xpert MTB/RIF assay^*a*^

tNGS	Xpert MTB/RIF		Total	χ^2^	*P* value
	MTB positive	Negative			
Positive	32	24	56	25.245	0.041
Negative	11	62	73		
Total	43	86	129		

^*a*^ McNemar’s test was used to calculate χ^2^ and the *P* value; *P* < 0.05 was considered statistically significant.

Using the 32 samples that were TB-positive under both tNGS and Xpert MIB/RIF, we further evaluated the two tests for the detection of mutations in the RRDR of *rpoB*. In total, RRDR mutations were detected in seven samples: six samples tested positive for mutations under tNGS, and four samples had fluorescence signals indicating mutations under Xpert MIB/RIF (Table 6). Three samples with mutations were detected by both tNGS and Xpert MIB/RIF. The three RRDR mutations were detected by tNGS alone. Furthermore, it was confirmed that the only RIF-positive sample detected by the Xpert MIB/RIF alone did not have a mutation in the RRDR.

**TABLE 6:** Comparison of tNGS- and Xpert MTB/RIF-based molecular testing for *rpoB* mutations (codons 426–452) in sputum specimens^*a*^

tNGS	Xpert MTB / RIF		Total	χ^2^	*P* value
	RIF-R signal	No RIF-R signal			
RRDR mutation	3	3	6	9.495	0.625
No RRDR mutation	1	25	26		
Total	4	28	32		

^*a*^ McNemar’s test was used to calculate χ^2^ and the *P* value; *P* < 0.05 was considered statistically significant.RIF-R, rifampicin resistance.

Next, using the WHO catalog of AMR-associated mutations, we analyzed AMR predictions for the 56 samples that were TB-positive under tNGS. The percentages of samples with mutations associated with the different antibiotic classes ranged from 5.4% to 96.4% (Table S4). Furthermore, among these 56 samples, there were relatively high frequencies of resistance-associated mutant genes of 96.4%, 35.7%, 26.8%, and 19.6%, in *rrs*, *rpoB*, *katG*, and *pncA*, respectively.

## DISCUSSION

Molecular testing methods are important tools for the detection of pathogenic members of the MTB complex. Although tNGS is an emerging testing technology, it has not yet gained clinical or commercial popularity in China. In this study, we systematically compared tNGS with Xpert MTB/RIF for the detection of MTB-specific nucleic acids and the *rpoB* mutation S450L in a laboratory strain of MTB. Using WGS as a standard, we confirmed the accuracy of tNGS. Furthermore, we performed a prospective comparative study of tNGS, Xpert MTB/RIF, culture, and smear microscopy using sputum samples from 129 patients with TB symptoms (50 in Beijing; 79 in Changsha). Our findings provide an assessment of tNGS as a valuable sequencing-based molecular testing method for the diagnosis of clinical TB and AMR. The tNGS assay simultaneously identified 158 MTB genes, including the 17,396 known AMR-associated loci. The minimum limit of MTB detection of tNGS (10^2^ CFU/mL) was slightly lower than that of the Xpert MTB/RIF assay (10^3^ CFU/mL). The previously reported detection limit of 131 CFU/mL for the Xpert MTB/RIF assay may be attributable to variability between laboratories (10). However, in MTB detection for clinical diagnosis, sputum samples are used and not a culture medium (as used in our study). The minimum limit of MTB detection, used here only as a reference, indicated that tNGS is comparable to Xpert for MTB detection.

Using a culture medium, the tNGS assay was revealed to have a higher limit of detection of RIF-associated mutations than the Xpert MTB/RIF assay. We also found that the minimum detection limit of the *rpoB* S450L mutation under tNGS (10^2^ CFU/mL) was 10^4^ times higher than that under Xpert MTB/RIF (10^6^ CFU/mL)—a significant difference. Despite the ability of Xpert MTB/RIF to detect MTB-specific nucleic acids at the range of 10^3^–10^5^ CFU/mL, no RIF mutations were detected using the manufacturer’s MTB/RIF test instructions. By contrast, the DNA specimens extracted from diluted MTB samples (10^2^–10^6^ CFU/mL) that were detectable by tNGS all carried the *rpoB* S450L mutation. Using WGS of the laboratory strain, we established the potential of the tNGS method for detecting the entire WHO catalog of 17,396 mutations associated with AMR. We concluded that tNGS as a molecular assay has greater application value than the Xpert MTB/RIF assay for the detection of RIF resistance and molecular prediction of resistance to other antimicrobial drugs.

Using clinical diagnostic criteria as a reference, the tNGS assay showed a level of sensitivity (48.6%) for MTB detection in clinical sputum samples that was similar to that of culture (46.8%) and higher than those of Xpert MTB/RIF (39.4%) and smear microscopy (34.9%). The goal of the Chinese government is to surpass 50% in etiological-positive detection for TB diagnosis including Xpert MTB/RIF, culture, and smear assays. Indeed, the detection rate in China is currently just reaching 50%. In the future, the application of the tNGS assay may contribute to the achievement of a higher goal.

In this evaluation, using clinical diagnostic criteria as a reference, the overall specificity of the tNGS assay was 85%; 20 patients had a negative clinical diagnosis of TB, but 3 of the 20 patient samples were positively detected by tNGS. We excluded the possibility of cross-contamination of these sputum samples by identifying the presence of MTB-specific DNA sequences, which included the RRDR region and loci associated with AMR, in the sequencing data from these three patients. These results suggested that the high sensitivity of tNGS lowers its specificity for TB diagnosis. Using tNGS results alone, we cannot diagnose suspected patients as active TB patients without imaging diagnosis or other assay results. However, the presence of MTB DNA sequences in sputum tested by tNGS assay has value as a diagnostic reference. The WHO guidelines state that any positive test result using Xpert MTB/RIF, culture, or smear microscopy is to be considered a bacteriologically confirmed diagnosis of TB (11). We recommend that the WHO standardize tNGS testing methods and provide guidance for tNGS-based diagnosis of MTB, thus allowing formal application of the tNGS assay as a pathogen-detection method in clinical practice.

The tNGS assay was an effective testing method for the smear- and culture-negative samples in this study. The current WHO guidelines state that the performance of tNGS assays may be less effective for smear-negative samples, suggesting that tNGS of smear-positive samples may be a more useful and cost-effective approach but only in the near term (12). However, of our 91 sputum samples with smear-negative results, MTB was detected by tNGS in 23 samples and by Xpert MTB/RIF in 11 samples. In addition, of 78 sputum samples with negative culture results, MTB was detected by tNGS in 17 samples and by Xpert MTB/RIF in three samples, with no identical samples. These findings indicated that molecular diagnostic methods, especially tNGS, were effective testing methods for samples in which the MTB concentration was too low to be detectable by smear or culture.

The tNGS assay has clear advantages over Xpert MTB/RIF for the detection of mutations associated with AMR. In terms of RRDR (*rpoB* codons 426–452) mutation detection (13), among 32 samples that were found to be TB positive by both Xpert MTB/RIF and tNGS, both methods detected mutations in three samples. One sample showing mutation detection by Xpert MTB/RIF alone was confirmed by sequencing to be a false-positive signal. Clear mutation loci information was identified for three samples with mutations that were only detected by tNGS. These results were consistent with the comparison of tNGS and Xpert MTB/RIF for the detection of the *rpoB* S450L mutation in the laboratory strain. Furthermore, our prediction analysis of additional AMR in 56 cases with tNGS-positive results may serve as a valuable reference for prescribing treatment in patients with TB.

The cost of tNGS assay in our study was USD 98 per sample. In China, the Xpert MTB/RIF is available as a commercial assay with a cost of USD 70–104 per sample. Thus, the cost of the two assays is similar. Notably, the manufacturer of GeneXpert MTB/RIF Ultra test cartridges announced in 2023 that the cartridges will be sold for USD 7.97 each to the Global Fund to Fight AIDS, TB, and Malaria, lending a cost advantage to Xpert MTB/RIF.

Notably, all of the experiments in this study were performed at the Beijing Center for Disease Control and Prevention, which has strict quality control and quality assurance procedures for TB diagnostic testing. However, there are some limitations to this study. First, the study population comprised only 129 participants from two cities in China, which may limit the generalizability of the results. Second, we did not include a comparison of tNGS and Xpert MTB/RIF assays in other laboratories. Third, using HIV-negative sputum samples only, we did not consider the impact of HIV infection on MTB detection using the tNGS and Xpert assays. In future studies, we plan to collect the sputum of HIV-positive and HIV-negative TB patients for comparative evaluation of MTB detection by the tNGS and Xpert MTB/RIF assays. The time required for tNGS (3 days) was much longer than that required for the Xpert MTB/RIF assay (2 hours) in this study. However, the value of tNGS is that it provides more accurate results and a larger number of AMR-associated gene mutations (14–16). Although Xpert MTB/RIF provided a more rapid diagnosis, our findings demonstrated that tNGS is suitable for correction of initial diagnosis, detection of AMR-associated gene mutations, prediction of AMR to drugs other than RIF, epidemiological characterization of MTB, and early warning of drug-resistant MTB. The application of tNGS-based drug-susceptibility testing (DST) could also improve DST, especially in laboratories that do not have the facilities and technical expertise to conduct other, more technically demanding phenotypic DST methods (17, 18).

## Data Availability

The raw sequence data reported in this paper have been deposited in the Genome Sequence Archive (Genomics, Proteomics & Bioinformatics 2021) at the National Genomics Data Center (Nucleic Acids Res 2022), China National Center for Bio-information/Beijing Institute of Genomics, Chinese Academy of Sciences (CRA013679), and are publicly accessible at https://ngdc.cncb.ac.cn/gsa.

## SUPPLEMENTAL MATERIAL

Table S1 spectrum.04098-23-s0001.docx The experiment design.

Table S2 spectrum.04098-23-s0002.xlsx Information on samples from 129 suspected tuberculosis patients.

Table S3 spectrum.04098-23-s0003.docx Comparison of targeted next generation sequencing (tNGS), Xpert MTB/RIF, and smear microscopy tests using culture testing of the 129 suspected tuberculosis patients as the reference.

Table S4 spectrum.04098-23-s0004.xlsx AMR predictions for the 56 samples that were TB-positive under tNGS.

ASM does not own the copyrights to Supplemental Material that may be linked to, or accessed through, an article. The authors have granted ASM a non-exclusive, world-wide license to publish the Supplemental Material files. Please contact the corresponding author directly for reuse.
