# Supplementary material for: Comparison of targeted next-generation sequencing and the Xpert MTB/RIF assay for detection of Mycobacterium tuberculosis in clinical isolates and sputum specimens
Source: Microbiol Spectr. 2024 Apr 11;12(5):e04098-23. doi: 10.1128/spectrum.04098-23 (PMC11064545; doi:10.1128/spectrum.04098-23)
Supplement: Table S1 — The experiment design. [file spectrum.04098-23-s0001.docx]

MTB isolates

Sanger

Xpert MTB/RIF

tNGS

WGS

10^5^ CFU/ml

10^4^ CFU/ml

10^3^ CFU/ml

10^2^ CFU/ml

10^1^ CFU/ml

Median

Low

Very low

-

10^6^ CFU/ml

10^5^ CFU/ml

10^4^ CFU/ml

10^3^ CFU/ml

10^2^ CFU/ml

10^1^ CFU/ml

MTB

MTB

MTB

MTB

MTB

10^6^ CFU/ml

Median

RIF-R

Not detected

not detected

not detected

-

RIF-R (rpoB S450L)

RIF-R (rpoB S450L)

RIF-R (rpoB S450L)

RIF-R (rpoB S450L)

RIF-R (rpoB S450L)

gene mutations associated with INH/SM/ETO

gene mutations associated with INH/SM/ETO

gene mutations associated with INH/SM/ETO

gene mutations associated with INH/SM/ETO

gene mutations associated with INH/SM/ETO

RIF-R (rpoB S450L)

RIF-R (rpoB S450L)

gene mutations associated with INH/SM/ETO

-

-

Sputum sample, n=129

Xpert MTB/RIF

tNGS

Culture

Smear

Positive，n=43

Negative, n=86

Positive，n=38

Negative, n=91

Positive, n=51

Negative, n=78

Positive, n=56

Negative, n=73

Mutation, n=43

No mutation，n=86

Fluorescent signal，n=5

No fluorescent signal，n=38

Table S1 Experimental design of the study

Y indicates positive identification of *Mycobacterium tuberculosis* (MTB); RIF, rifampicin; Sanger, Sanger sequencing; tNGS, targeted next generation sequencing; INH, isoniazid; SM, streptomycin; ETO, ethionamide.
