# Supplementary material for: Comparison of targeted next-generation sequencing and the Xpert MTB/RIF assay for detection of Mycobacterium tuberculosis in clinical isolates and sputum specimens
Source: Microbiol Spectr. 2024 Apr 11;12(5):e04098-23. doi: 10.1128/spectrum.04098-23 (PMC11064545; doi:10.1128/spectrum.04098-23)
Supplement: Table S3 — Comparison of targeted next generation sequencing (tNGS), Xpert MTB/RIF, and smear microscopy tests using culture testing of the 129 suspected tuberculosis patients as the reference. [file spectrum.04098-23-s0003.docx]

Table S3 Comparison of targeted next generation sequencing (tNGS), Xpert MTB/RIF, and smear microscopy tests using culture testing of the 129 suspected tuberculosis patients as the reference

| Method | Culture | | sensitivity(%)  (95% CI) | Specificity(%)  (95% CI) | Pvalue |
| --- | --- | --- | --- | --- | --- |
|  | Positive | Negative |  |  |  |
| tNGS |  |  | 76.5(39/51)  (62.2-86.8) | 78.2(61/78)  (67.1-86.4) | 0.458 |
| Positive | 39 | 17 |  |  |  |
| Negative | 12 | 61 |  |  |  |
| Xpert MTB / RIF |  |  | 78.4(40/51)  (64.3-88.2) | 96.2(75/78)  (88.4-99.0) | 0.057 |
| Positive | 40 | 3 |  |  |  |
| Negative | 11 | 75 |  |  |  |
| Smear |  |  | 72.5（37/51)  (58.0-83.7) | 98.7（77/78)  (92.1-99.9) | 0.001 |
| Positive | 37 | 1 |  |  |  |
| Negative | 14 | 77 |  |  |  |

MTB/RIF, *Mycobacterium tuberculosis*/rifampicin.
